# Evaluation of Lower Urinary Tract Symptoms in Males and Urinary Incontinence in Females in Primary Health Care in Greece

**DOI:** 10.3390/medicina60030389

**Published:** 2024-02-25

**Authors:** Claire Gkatzoudi, Izolde Bouloukaki, Charalampos Mamoulakis, Christos Lionis, Ioanna Tsiligianni

**Affiliations:** 1 Department of Social Medicine, School of Medicine, University of Crete, 71410 Heraklion, Greece; gkatzoudiclaire@yahoo.gr (C.G.); lionis@uoc.gr (C.L.); i.tsiligianni@uoc.gr (I.T.); 2 Department of Urology, University General Hospital of Heraklion, Medical School, University of Crete, 71003 Heraklion, Greece; mamoulak@uoc.gr

**Keywords:** LUTS, ED, UI, primary care, screening, quality of life

## Abstract

*Background and Objectives*: The significant prevalence of Lower Urinary Tract Symptoms (LUTS), erectile dysfunction (ED), and associated adverse effects calls for increased attention in primary care settings. In Greece, there is a lack of sufficient data for LUTS and ED screening in primary care. Therefore, the aim of our study was to estimate the prevalence of LUTS and ED, identify associated risk factors, and evaluate their impact on quality of life among adult primary healthcare users aged 40 years and older in Crete, Greece. *Materials and Methods*: A cross-sectional study was conducted to explore the prevalence of LUTS and ED in 1746 primary health care users visiting rural primary health care practices in Crete, Greece. Participants underwent a comprehensive evaluation including demographic parameters, screening for LUTS utilizing the validated International Prostate Symptoms Score (IPSS) questionnaire and for ED using the International Index of Erectile Function (IIEF-5), in males, and for urinary incontinence in women with the International Consultation on Incontinence Questionnaire–Urinary Incontinence Short Form (ICIQ-UI SF). Participants with a prior diagnosis of LUTS or ED were excluded (n = 183). *Results*: Out of 536 participants finally included (n = 1746 screened), 32% of males and 36% of females exhibited moderate to severe LUTS. Following adjustments, we identified advanced age, retirement, and the presence of diabetes type 2 as factors associated with the occurrence of LUTS in men. Patients with LUTS also had a substantially increased likelihood of experiencing ED. Moreover, it was observed that women with hypertension or diabetes type 2 and lower education levels face an increased likelihood of developing LUTS, which adversely affects their quality of life. *Conclusions*: In conclusion, the findings of this study reveal a high occurrence of LUTS and ED in adults aged 40 years and older who utilize primary healthcare services, with a negative impact on their quality of life.

## 1. Introduction

Non-neurologic Lower Urinary Tract symptoms (LUTS) encompass a wide range of symptoms, such as storage, voiding, and incontinence symptoms, with the majority of patients presenting a combination of these symptoms [1]. These symptoms are more prominent and have a tendency to worsen as individuals age [2]. According to a recent meta-analysis that examined the prevalence of LUTS worldwide, approximately 63.2% of individuals in the general population had any type of LUTS, and 31.3% experienced symptoms of moderate-to-severe intensity [3].

LUTS are usually not life-threatening but can interfere with daily functioning, socialization [4], and mental well-being [5], thereby adversely affecting an individual’s health-related quality of life (HRQOL) [6]. More specifically, research conducted in the USA, the UK, and Sweden revealed that LUTS were linked to increased levels of anxiety and depressive symptoms as well as decreased HRQOL [7]. In a recent study, latent class analysis was employed to categorize LUTS among primary care patients into distinct classes and then compare the HRQOL differences between these classes [8]. The study revealed that a significant proportion of primary care patients (nearly 75%) experienced varying degrees of LUTS. Six distinct LUTS classes were identified, including “asymptomatic” (26.0%), “mild symptoms” (22.6%), “moderate multiple symptoms” (17.0%), “urgency symptoms” (13.8%), “urinary incontinence” (12.0%), and “severe multiple symptoms” (8.6%). Differences were found in the gender distribution and prevalence of the cardiovascular disease across classes, with patients experiencing “severe multiple symptoms” and “urinary incontinence” reporting the lowest HRQOL. It is also crucial to highlight that LUTS can have a hidden neurological cause in patients without a formally diagnosed neurological disorder (“occult neurology”) [9] or hide another underlying medical condition like cardiovascular disease or diabetes type 2 [10].

In males, a variety of symptoms originating from disorders and diseases impacting the bladder, prostate, and urethra collectively fall under the umbrella term LUTS [11,12]. They are bothersome and increase the risk of falls [13] and psychological distress, negatively affecting quality of life [7], while also imposing a substantial economic burden on patients and healthcare systems [14,15]. Although there is a wide range of conditions that can primarily manifest as LUTS, traditionally, LUTS in males have primarily been associated with benign prostatic hyperplasia (BPH) [16]. Importantly, LUTS secondary to BPH (BPH-LUTS) has been acknowledged as an independent risk factor for ED [17], further exacerbating the decline in HRQOL for these patients. It appears that LUTS, particularly nocturia, manifest prior to erectile dysfunction (ED) in a 15-year longitudinal study conducted on elderly men [17].

In females, LUTS are also prevalent, with a significant number of women experiencing symptoms of urinary incontinence (UI). Urinary incontinence is characterized by the involuntary leakage of urine. The prevalence of this condition increases with age, ranging from 17% in women over 20 years old to 38% in those aged 60 years or older [18,19,20]. Merely 25% of women experiencing UI actively pursue medical attention, and the postponement of seeking treatment can extend to multiple years [21]. Untreated UI is correlated with falls, fractures, depression, and sleep disturbance [22,23,24]. These symptoms have a profound impact on individuals and society, causing distress, embarrassment, and significant costs [25].

LUTS are anticipated to be initially assessed and managed in primary care settings. Nonetheless, the assessment of LUTS and the implementation of conservative measures in primary care are limited and inconsistent [26,27]. Indeed, the level of adherence to recommended standards of care for LUTS among men in primary care seems to be low, particularly among older men who have a higher prevalence of LUTS [26]. Among women experiencing LUTS, only 23% receive any form of treatment in primary care [21]. Therefore, in order to address suboptimal LUTS management, heightened attention from primary care physicians is expected. Consequently, active screening for LUTS in primary care should be considered in order to identify patients exhibiting symptoms that could benefit from treatment, resulting in improvements in quality of life, decreased long-term morbidity, and potential savings in medical resources [28]. However, it is crucial for primary care physicians to have practical resources that can aid in the assessment of LUTS and improve patient involvement in lifestyle changes and conservative (non-surgical) management interventions [27]. Symptom questionnaires are standard instruments that can be utilized for assessing male and female LUTS, identifying symptom variations, and continuously monitoring treatment effectiveness [29,30].

In Greece, there are limited data available for LUTS screening in primary care [31,32,33]. Therefore, our study aimed to assess the prevalence of LUTS, specifically symptoms of benign prostatic hyperplasia (BPH) and erectile dysfunction in men and UI in women, in a primary health care center in Greece. Additionally, we aimed to analyze the association between LUTS and other long-term diseases and their influence on the quality of life of these individuals.

## 2. Materials and Methods

### 2.1. Study Participants

A cross-sectional study employing a quantitative survey was conducted to examine the prevalence of LUTS in 1738 primary health care users visiting eight rural primary health care practices in Moires, Crete, Greece, from January to September 2021. Participants were required to meet the following criteria to be included: (a) be aged ≥40 years, and (b) be able to provide written informed consent. Participants were excluded if they refused to participate, had severe neurological or mental disease, malignancy, severe cardiovascular disease, were pregnant, or had a poor understanding of the Greek language.

### 2.2. Data Collection

Participants underwent a comprehensive evaluation that assessed demographic parameters, such as age and gender, comorbidities (including previous physician-based diagnosis for BPH, ED, or UI), level of education, and work status (including the presence of manual work). The male participants underwent initial screening for LUTS utilizing the validated International Prostate Symptoms Score (IPSS) questionnaire. The International Index of Erectile Function (IIEF-5) was utilized for the assessment of ED in males. For the evaluation of urinary incontinence in women, the International Consultation on Incontinence Questionnaire–Urinary Incontinence Short Form (ICIQ-UI SF) was implemented. Each participant’s evaluation lasted roughly 20 to 30 min. The questionnaires employed and interviews conducted were all conducted in the Greek language. Participants completed and responded to all the questionnaires based on their symptoms during the interview.

### 2.3. Study Tools and Outcomes

#### 2.3.1. The International Prostate Symptom Score (IPSS)

The IPSS is an eight-item questionnaire widely employed for the screening, diagnosis, and monitoring of symptoms linked to benign prostatic hyperplasia and LUTS [34,35]. The seven items of the IPSS were employed to assess LUTS in males, specifically incomplete bladder emptying, frequency, intermittency, urgency, weak stream, straining to void, and nocturia. The answers are given points on a scale of 0 to 5. The overall score ranges from 0 to 35, representing the spectrum from asymptomatic to highly symptomatic. A score that is equal to or greater than 8 indicates the presence of clinically significant LUTS. The IPSS values were classified as mild (scores 0–7), moderate (scores 8–19), and severe (scores 20–35) LUTS. Furthermore, the IPSS incorporates an 8th question, a quality-of-life assessment, in the form of a single 7-point scale question, inquiring about the patient’s hypothetical feelings if they were to live with their current urinary condition for the rest of their life. The Likert scale was used to measure the quality-of-life score, ranging from 0 (delighted), 1 (pleased), 2 (mostly satisfied), 3 (mixed about equally satisfied and dissatisfied), 4 (mostly dissatisfied), 5 (unhappy) and 6 (terrible). The IPSS questionnaire has been translated and validated for the purpose of assessing LUTS in the Greek population [36].

#### 2.3.2. International Index of Erectile Function

The IIEF-5 is a simplified and standardized questionnaire employed to diagnose and evaluate the severity of ED [37]. The IIEF-5 questionnaire consists of five questions, each of which can be scored from 0 or 1 (representing the worst) to 5 (representing the best). The final score ranges from 1 to 25 points, with a lower score indicating a deterioration in erectile function and a score ≤ 21 confirming the presence of erectile dysfunction. Furthermore, ED is classified based on the overall score as severe (score 0–7), moderate to severe (score 8 to 11), mild to moderate (score 12 to 16), mild (score 17 to 21), and absent (score 22 to 25). The IIEF-5 questionnaire has been translated and validated for the purpose of assessing ED in the Greek population [38].

#### 2.3.3. International Consultation on Incontinence Questionnaire–Urinary Incontinence Short Form (ICIQ-UI SF)

ICIQ-UI SF is a questionnaire that is relatively concise, composed of six items, encompassing two demographic items and three items for rating symptoms experienced in the past 4 weeks (frequency of UI episodes, amount of leakage, and overall impact of UI). The total score obtained from these three items corresponds to the ICIQ-UI-SF score, which has a range of 0 to 21 points. A higher score indicates a greater severity of symptoms and a more significant impact on the quality of life for women [39]. The sixth item corresponds to the self-diagnostic question concerning the type of UI and is not taken into account when calculating the final score. The ICIQ-UI SF questionnaire has undergone translation and validation for the specific purpose of evaluating UI within the Greek population [40].

### 2.4. Procedure

The participants who were included in the study were provided with information about the study’s objectives during their visit. Following their agreement to participate, they proceeded to submit written consent and anonymously complete the questionnaires. No compensation was given for their participation. In an effort to minimize the influence of social desirability bias, participants were provided with guidance to place their completed study materials into a non-transparent container situated outside the office.

This study adhered to the guidelines specified in the Declaration of Helsinki and received approval from the University of Crete Research Ethics Committee (REC-UOC) (Protocol Number: 141/11 July 2019). Written, informed consent was obtained from all subjects.

### 2.5. Statistical Analysis

Our analysis was restricted to only those participants who had completed all the questionnaires. In the case of continuous variables with a normal distribution, the results are reported as the mean ± standard deviation (SD); conversely, for variables without a normal distribution, the median (25–75th percentile) was employed. The presentation of qualitative variables involves the use of an absolute numerical value, usually expressed as a percentage. To conduct comparisons between groups, we utilized a two-tailed *t*-test for independent samples (when data followed a normal distribution) or a Mann–Whitney U-test (when data did not follow a normal distribution) for continuous variables. Furthermore, the chi-square test was employed for categorical variables. Binomial logistic regression was employed to analyze the factors associated with LUTS and ED while accounting for various potential explanatory variables, including age, gender, presence of chronic disease, work status, and level of education. Multicollinearity among the predictors was assessed using collinearity statistics to ensure that the collinearity between predictor variables was within the acceptable range, as indicated by the tolerance value variance inflation factor. Results were deemed significant if the *p*-values were less than 0.05. Data were analyzed using SPSS software (version 25, SPSS Inc., Chicago, IL, USA).

## 3. Results

### 3.1. Patient Characteristics

**Figure 1:**
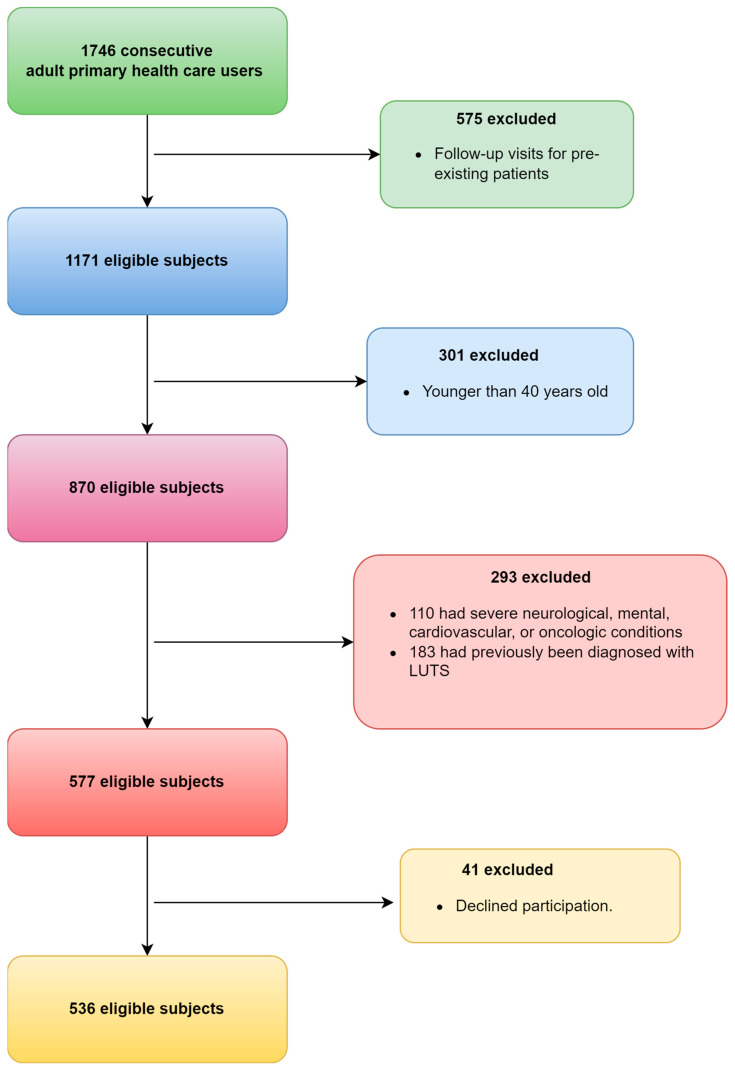
The flowchart of patients that were finally included.

A total of 1746 consecutive adult primary health care users visiting eight primary health care practices, regardless of their reason for visit, were invited to participate in the study. Based on the inclusion criteria, a total of 1210 patients were excluded (Figure 1). Of the patients excluded, 575 were follow-up visits from existing patients, and 301 were younger than 40 years old. Out of the remaining 870 eligible subjects, 110 (12%) had severe neurological, mental, cardiovascular, or oncologic conditions, while 183 (21%) had previously received a diagnosis of LUTS, and 41 (5%) declined to participate.

The final study population involved 536 participants, with a male representation of 206 (38%). Participants’ ages varied between 40 and 97 years; 125 (23%) were aged 40–50, 150 (28%) were aged 51–64, and 261 (49%) were aged ≥65 years. A substantial portion (71%) of patients had educational backgrounds exceeding primary school, and almost half (48%) were retired, with a median retirement period of 10 (5, 15) years. In the context of chronic diseases, the prevalence of at least one chronic disease was observed in 346 patients (65%). Table 1 provides a further description of the socio-demographic characteristics and health status factors of the 536 participants.

### 3.2. LUTS in Male Population

The IPPS questionnaire was completed by 206 out of 219 male participants, resulting in a response rate of 94%. Total IPSS scores ranged between 0 and 28, with a median score of 3 (0, 9) (Table 2).

**Figure 2:**
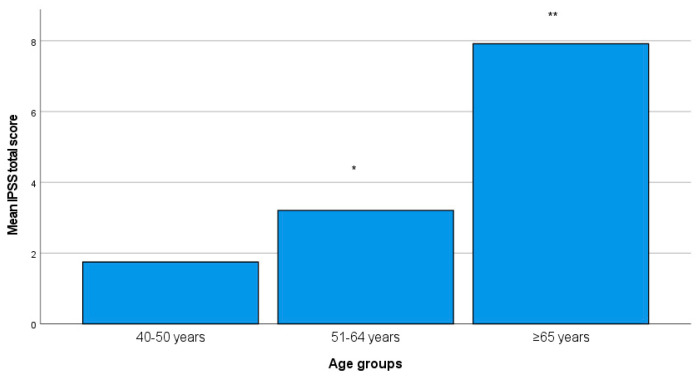
Comparisons of IPSS among various age groups. Compared with that in the group aged 40–50 years old, * *p* < 0.001; compared with that in the group aged 51–64 years old, ** *p* < 0.001.

A considerable proportion of patients (n = 115, 56%) had at least one symptom of LUTS (IPPS score ≥ 1). A total of 139 (68%) reported mild symptoms, while 91 (44%) were identified as asymptomatic (IPPS score: 0). IPSS scores varied significantly among different age groups, as shown in Figure 2. The prevalence of moderate to severe LUTS (defined as IPSS ≥ 8) was found to be as high as 82% among participants aged 60 years or older.

The group of men with moderate-to-severe LUTS (IPPS score ≥ 8, 32%) was characterized not only by advanced age but also by lower education and lower rates of manual work when compared to their non-LUTS counterparts. Hypertension, diabetes type 2, atrial fibrillation, and hyperlipidemia were all found to be more prevalent in men with LUTS; however, there were no statistically significant differences between the LUTS and non-LUTS groups in terms of COPD/asthma, depression, or rheumatologic disease. Table 3 provides an overview of the detailed baseline characteristics.

Following adjustment for confounding factors, the presence of moderate-to-severe LUTS was found to be independently associated with older age (OR = 1.076, 95% CI 1.039–1.114; *p* < 0.001), retirement (OR = 4.010, 95% CI 1.310–12.281; *p* = 0.015), the presence of at least one chronic disease (OR = 2.313, 95% CI 1.082–4.943; *p* = 0.03), and diabetes type 2 (OR = 5.121, 95% CI 2.125–12.338; *p* < 0.001). Furthermore, it is worth noting that 25% of the participants experienced a decline in their quality of life (IPSS quality of life score ≥ 3) as a consequence of urinary symptoms (Table 2), and this was found to have a strong independent correlation with the IPPS score (OR = 1.404, 95% CI 1.256–1.569).

### 3.3. Erectile Dysfunction in Male Population

The IIEF-5 questionnaire was completed by 199 out of 219 male participants, yielding a response rate of 91%. Total IIEF-5 scores ranged between 0 and 25, with a median score of 10 (4, 20) (Table 2). A significant percentage of patients (84%) indicated at least mild ED (IIEF-5 score ≤ 21), whereas 63% indicated moderate to severe ED. The group of men with moderate-to-severe ED was characterized by advanced age, a lower level of education, and higher retirement rates when compared to their non-ED counterparts. There were no statistically significant differences between the ED and non-ED groups in terms of chronic diseases, including hypertension, atrial fibrillation, COPD/asthma, diabetes type 2, depression, hyperlipidemia, or rheumatologic disease. The detailed baseline characteristics are summarized in Table 4.

Following adjustment for confounding factors, the presence of moderate-to-severe ED was found to be independently associated with the age of ≥60 years (OR = 2.248, 95% CI 1.042–5.658; *p* = 0.040) and retirement (OR = 6.949, 95% CI 2.475–19.505; *p* < 0.001).

**Figure 3:**
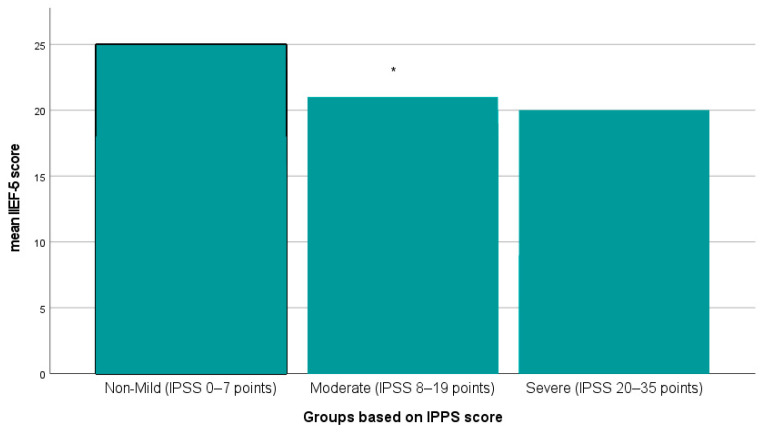
Comparisons of IIEF-5 among groups based on IPPS. Compared with that in the non-mild IPPS group, * *p* < 0.001.

It is important to highlight that the IIEF score was greatly influenced by the severity of LUTS (Figure 3). Furthermore, patients with LUTS (moderate to severe IPPS) had a significantly higher prevalence of ED, with 76% compared to 55% observed by those without LUTS (*p* = 0.006). The presence of LUTS was found to be the most significant predictor of erectile dysfunction (OR = 2.393, 95% CI 1.091–5.249; *p* = 0.030), followed by age ≥60 years (OR = 1.731, 95% CI 0.918–3.263; *p* = 0.040).

### 3.4. LUTS in Female Population

The ICIQ-UI-SF was completed by 342 out of 358 female participants, resulting in a response rate of 96%. Total ICIQ questionnaire scores ranged between 0 and 28, with a median score of 3 (0, 8) (Table 2). A considerable proportion of participants (51%) reported slight to moderate symptoms, while 142 (42%) were identified as asymptomatic (ICIQ score: 0). The group of females with moderate-to-severe LUTS (36%) was characterized by advanced age, lower education, and higher unemployment rates when compared to their non-LUTS counterparts. Hypertension, diabetes type 2, and hyperlipidemia were all found to be more prevalent in females with LUTS; however, there were no statistically significant differences between the LUTS and non-LUTS groups in terms of COPD/asthma, depression, atrial fibrillation, or rheumatologic disease. Table 5 provides an overview of the detailed baseline characteristics.

Following adjustment for confounding factors, the presence of moderate-to-severe LUTS was found to be independently associated with lower education (OR = 2.738, 95% CI 1.309–5.730; *p* = 0.007), the presence of at least one chronic disease (OR = 2.387, 95% CI 1.334–4.271; *p* = 0.003), hypertension (OR = 1.763, 95% CI 1.004–3.094; *p* = 0.048), and diabetes type 2 (OR = 2.873, 95% CI 1.434–5.758; *p* = 0.003). Furthermore, it is worth noting that 31% of the participants experienced a decline in their quality of life as a consequence of urinary symptoms (Table 2), and this was found to have a strong independent correlation with the ICIQ-UI-SF score (OR = 2.329, 95% CI 1.858–2.920, *p* < 0.001).

## 4. Discussion

The aim of our study was to analyze the prevalence of LUTS, UI, and ED, identify associated risk factors, and evaluate their impact on quality of life among 536 finally included out of 1746 initially screened adult primary healthcare users aged 40 years and above in Greece. The results showed that 32% of males and 36% of females exhibited clinically significant LUTS and UI, respectively. Following adjustments, we identified advanced age, retirement, and the presence of diabetes type 2 as factors associated with the occurrence of LUTS in men. Moreover, a significant positive association was identified between LUTS in males and the presence of ED, thereby having considerable implications for the quality of life of these patients. On the other hand, women with hypertension, type 2 diabetes, and lower education were found to be at a higher risk for developing UI, which in turn negatively impacts their quality of life.

The prevalence of LUTS has been assessed through multiple large-scale population-based studies conducted in various geographical regions across the globe. These studies consistently indicate a high prevalence of LUTS among males aged ≥40 years globally (62–73%), with rates ranging from 6 to 34% for moderate to severe LUTS [41,42,43,44,45]. According to our study, the overall prevalence rate of LUTS is slightly lower compared to previous research (56%). However, there is a higher prevalence compared to the average global prevalence of moderate to severe symptoms (32%). This difference is particularly evident when comparing it to previous studies conducted in primary care [46,47] settings. Our study found that the overall prevalence of moderate to severe LUTS within the age range of 40 years and above was 32%, whereas a prevalence of 26.4% was observed in 549 males attending primary care of the same age range who completed the same questionnaire [47]. Furthermore, the prevalence of moderate to severe LUTS, also based on the IPSS questionnaire, was 20.5% among 450 males aged ≥60 years in primary care settings, while our male participants within the same age range had a much higher prevalence of 82% [46]. To our knowledge, there has been no study conducted in Greece to assess the prevalence of LUTS in the male population within primary care settings. An earlier survey with 132 male participants aged ≥40 years in a hospital setting in Greece revealed a slightly higher prevalence of moderate to severe LUTS, at 37%, compared to the 32% prevalence observed in our population within the same age range [48].

The increased prevalence of moderate-to-severe LUTS in our study can likely be attributed to the low rate of seeking medical guidance for LUTS among the rural population attending our primary care center. This is indeed further supported by our data, which show that only 183 out of 870 (21%) participants aged ≥40 years of our population have received a formal diagnosis of LUTS, while 115 out of 206 (56%) of the male population included without a previous diagnosis of LUTS experienced at least one LUTS based on the screening with the IPPS questionnaire. Earlier studies have also suggested that, despite the widespread prevalence of LUTS globally and its significant link to major health problems in males, there is a consistent pattern of low seeking medical consultation rates, limited to advanced disease stages [49]. The exploration of factors affecting men’s decision to seek treatment for LUTS has primarily been conducted within tertiary healthcare settings [50,51]. There are multiple factors that have been identified, such as a lack of knowledge about LUTS, perceiving LUTS as a natural part of aging, the severity of symptoms and how they affect the quality of life, advice from others and the media, and feelings of embarrassment [50,51,52]. However, there are limited data regarding factors that prompt early medical intervention in primary care settings. Findings from primary care studies indicated that respondents commonly considered LUTS as a “natural consequence of aging” and were hesitant to talk about sexual or urinary problems [53,54,55].

In our study, there were significant associations between the presence of moderate-to-severe LUTS in males and factors such as older age, retirement, and the presence of at least one chronic disease, particularly type 2 diabetes. These findings are consistent with previous studies, which have shown that LUTS becomes more prevalent as individuals age [16,56]. They also suggest that comorbid conditions like type 2 diabetes could be potential targets for prevention, presenting an opportunity for intervention [57,58,59,60]. Identifying LUTS early on is indeed crucial to prevent potential complications that can impact general health, psychological well-being, depression, stress levels, and even sexual satisfaction and desire [7,61,62,63]. Within our population, a quarter of the participants experienced a decrease in their quality of life due to LUTS.

Individuals who exhibit clinically significant LUTS are also at an elevated risk of developing ED [64,65,66,67]. Our study revealed that LUTS increased the likelihood of developing ED by 2.3 times, even when adjusting for age, gender, education, or comorbidities. This relationship has been extensively investigated, providing evidence for the causal relationship between LUTS and ED [68]. The prevalence of moderate-to-severe ED was 63% within our population, rising to 84% in patients experiencing at least mild ED. The recorded prevalence of at least mild ED ranges from 63 to 68% in studies that employ the same screening questionnaire for ED in primary care settings [69] and in community-based research [70]. However, in primary care, symptoms of ED are often not fully disclosed due to various factors such as reporting bias, cultural influences, physicians’ failure to inquire about patients’ sexual health, and the social stigma surrounding the condition [71]. Nevertheless, the percentage of participants who completed the IIEF-5 questionnaire was 91%, indicating a high response rate when compared to previous research [72,73]. This might inspire physicians to encourage patients to openly discuss their sexual health [74].

It is noteworthy that women also commonly experience LUTS, with urinary incontinence being two to four times more prevalent in females than in males across all age groups [75]. Assessing the prevalence of UI in women within the general population and primary care continues to present difficulties, as evidenced by existing studies that indicate UI rates ranging from 5% to 69% [76,77,78,79]. The findings of a recent study indicate that the overall prevalence of UI in adult women in the United States was 62%, reflecting a notable increase from previous estimates of 38–53% [20,76,80,81]. In Greece, the prevalence rates of UI fall within the range of 27–44% [31,32,33], which is comparable to our prevalence rate of 36%. The variations in prevalence rates among European countries, the US, and Greece can be explained by differences in how surveys are conducted, data are collected, UI is defined, study populations are selected, age groups of respondents are considered, and cultural or ethnic factors are considered. Among the associated factors of UI in our population, diabetes type 2 had the highest odds ratio for predicting UI. There is evidence from a previous study conducted in Greece that supports this, indicating that diabetes was associated with moderate/severe LUTS in women, while no such association was found in men [36]. In addition, it has been observed that a minority of women with UI are actively seeking medical consultation for their condition [31], likely attributable to underestimating the condition and to a high level of embarrassment with UI [82]. Consequently, affected individuals tend to deny and conceal UI, thereby experiencing restrictions on their physical and psychosocial well-being and enjoyment of life. Specifically, the major impacts encompass decreased self-esteem and isolation from social circles, alongside additional unfavorable consequences such as increased anxiety, depression, impaired sexual well-being, decreased physical activity levels, and overall poor quality of life [83,84]. The impact on quality of life was evident in our study as well, with 31% of the female participants reporting a decrease in their quality of life due to urinary symptoms. Given the results presented in this study, it is apparent that the considerable prevalence of LUTS and ED and their impact on quality of life cannot be overlooked.

These observations underline the importance of conducting LUTS screening and diagnosis but also draw attention to the limited availability of these actions in primary healthcare settings. Within primary health care, healthcare providers have the potential to identify symptoms and provide health education strategies that may prevent or address a wide range of health issues, with the goal of enhancing overall health-related quality of life. However, due to the limited provision of LUTS screening in primary care, it is crucial to educate primary care health professionals. This education will empower them to effectively evaluate LUTS and raise awareness about the condition among the population, encouraging them to pursue medical treatment. In addition, primary care practices could start implementing a screening program for LUTS led by practice nurses during initial consultations. GPs, once adequately trained, could also then provide the best treatment options. Implementing this approach would prevent the possibility of overlooking treatment options for LUTS [85].

To the best of our knowledge, this study represents one of the few studies assessing LUTS and ED in a primary care population in Greece, a population with its own distinct socio-demographic and cultural characteristics. Among the notable strengths of the study are the inclusion of a relatively large sample size of adult men and women with a high response rate, as well as the use of previously tested and validated instruments to assess the presence of LUTS and ED. However, it is important to acknowledge a few limitations of this research. One limitation of the study design is that the cross-sectional design is unable to establish causal connections between LUTS, ED, and associated factors. Another limitation of the study is that it did not evaluate the presence of obstructive sleep apnea syndrome (OSAS), a condition that is known to be associated with the severity of LUTS [86], previous occupational activities [87], or medications that could potentially impact LUTS [88,89]. Additionally, the participants in the study were only recruited from rural primary care centers, which might not accurately reflect the overall population in Greece. Nonetheless, the data gathered from this study prove to be a valuable tool in enhancing our knowledge of the frequency of LUTS and ED in primary care, especially considering the limited information available in our country. Finally, we conducted our study during the third surge of the COVID-19 pandemic, which could have impacted the sample size as a result of fewer in-person appointments. Future large-scale studies may, therefore, need to consider these factors.

## 5. Conclusions

In conclusion, the findings of this study reveal a high occurrence of LUTS and ED in adults aged 40 years and older who utilize primary healthcare services. As LUTS and ED have a negative impact on an individual’s quality of life, it is crucial to highlight the role of primary care providers in screening for these conditions. By screening for these symptoms, it is possible to identify patients exhibiting symptoms that could benefit from treatment, resulting in improvements in their overall quality of life. Future studies evaluating a larger number of patients from different regions of Greece are warranted.

## Figures and Tables

**Table 1:** Characteristics of the participants (n = 536).

Characteristics	
**Age (years)**	63 ± 13
Age group 40–50 years	125 (23%)
Age group 51–64 years	150 (28%)
Age group ≥65 years	261 (49%)
**Gender, male (%)**	206 (38%)
**Education level (n = 512)**	
Primary level	131 (25%)
Secondary level	224 (44%)
Higher level	157 (31%)
**Occupational status**	82 (9%)
Retired	257 (48%)
Sedentary work	161 (30%)
Manual work	118 (22%)
**Comorbidities**	
Arterial hypertension	255 (48%)
COPD/asthma	46 (9%)
Diabetes type 2	93 (17%)
Atrial Fibrillation	32 (6%)
Depression (on medication)	41 (8%)
Rheumatologic disease	42 (8%)
Hyperlipidemia	184 (34%)

COPD: Chronic Obstructive Pulmonary Disease.

**Table 2:** Assessment of LUTS and erectile dysfunction (ED) using questionnaires in the study population.

Symptoms	
Male population	
**IPSS score (range 0–35)** **(n = 206)**	3 (0, 9)
Non-Mild (IPSS 0–7 points)	139 (68%)
Moderate (IPSS 8–19 points)	64 (31%)
Severe (IPSS 20–35 points)	3 (1%)
**IPSS quality of life score (range 0–** **6)**	2 (0, 3)
Poor quality of life (score ≥3)	46 (25%)
**IIEF-5 (range: 0–25 points)** **(n = 199)**	10 (4, 20)
Normal (IIEF-5 22–25 points)	32 (16%)
Mild (IIEF-5 17–21 points)	42 (21%)
Mild to moderate (IIEF 12–16 points)	22 (11%)
Moderate to severe (IIEF 8–11 points)	22 (11%)
Severe (IIEF-5 0–7 points)	81 (41%)
**Female population**	
**ICIQ-UI-SF score (range 0–21)**	3 (0, 8)
ICIQ 0	142 (41.7%)
ICIQ slight (1–5),	75 (22%)
ICIQ moderate (6–12),	100 (29%)
ICIQ severe (13–18)	24 (7%)
ICIQ very severe (19–21)	1 (0.3%)
**Quality of life due to urinary symptoms total score**	1 (0, 3)
**Poor quality of life (score ≥3)**	106 (31%)

LUTS: Lower Urinary Tract Symptoms; IPPS: International Prostate Symptom Score; IIEF-5: International Index of Erectile Function; ICIQ-UI-SF: International Consultation on Incontinence Questionnaire–Urinary Incontinence Short Form.

**Table 3:** Demographic and clinical characteristics of the study subjects according to LUTS in males (n = 206).

Characteristics			
	**Non-LUTS**	**LUTS**	***p*-Value**
	None–mild IPSS	Moderate–severe IPSS	
	n = 139	n = 67	
**Age (years)**	58 ± 12	70 ± 11	0.001
**Age groups**			<0.001
Age group 40–50 years	52 (37%)	4 (6%)	
Age group 51–64 years	42 (30%)	11 (16%)	
Age group ≥65 years	45 (33%)	52 (78%)	
**Education level**			<0.001
Primary level	17 (13%)	23 (35%)	
Secondary level	64 (48%)	28 (43%)	
Higher level	52 (39%)	14 (22%)	
**Occupational status**			<0.001
Retired	37 (27%)	50 (75%)	
Sedentary work	38 (27%)	7 (10%)	
Manual work	64 (46%)	10 (15%)	
**Comorbidities**			
Arterial hypertension	52 (37%)	43 (64%)	<0.001
COPD/asthma	12 (9%)	9 (13%)	0.286
Diabetes type 2	13 (9%)	25 (37%)	<0.001
Atrial Fibrillation	7 (5%)	9 (13%)	0.035
Depression (on medication)	3 (2%)	5 (8%)	0.065
Rheumatologic disease	4 (3%)	2 (3%)	0.966
Hyperlipidemia	44 (32%)	35 (52%)	0.004

LUTS: Lower Urinary Tract Symptoms; COPD: Chronic Obstructive Pulmonary Disease.

**Table 4:** Demographic and clinical characteristics of the study subjects according to ED in males (n = 199).

Characteristics			
	**Non-ED**	**ED**	***p*-Value**
	None–mild IIEF-5	Moderate–severe IIEF-5	
	n = 74	n = 125	
**Age (years)**	59 ± 9	63 ± 15	0.001
**Age groups**			<0.001
Age group 40–50 years	9 (12%)	47 (38%)	
Age group 51–64 years	45 (61%)	8 (6%)	
Age group ≥65 years	20 (27%)	71 (56%)	
**Education level**			0.028
Primary level	11 (15%)	28 (23%)	
Secondary level	43 (58%)	47 (38.5%)	
Higher level	20 (27%)	47 (38.5%)	
**Occupational status**			<0.001
Retired	16 (22%)	65 (52%)	
Sedentary work	21 (28%)	23 (18%)	
Manual work	37 (50%)	37 (30%)	
**Comorbidities**			
Arterial hypertension	28 (38%)	60 (48%)	0.163
COPD/asthma	8 (10%)	12 (10%)	0.784
Diabetes type 2	12 (16%)	25 (20%)	0.507
Atrial Fibrillation	3 (4%)	12 (10%)	0.152
Depression (on medication)	2 (3%)	6 (5%)	0.467
Rheumatologic disease	3 (4%)	2 (2%)	0.285
Hyperlipidemia	26 (35%)	49 (39%)	0.567

ED: Erectile Dysfunction; COPD: Chronic Obstructive Pulmonary Disease.

**Table 5:** Demographic and clinical characteristics of the study subjects according to LUTS in females (n = 342).

Characteristics			
	**Non-LUTS**	**LUTS**	***p*-Value**
	None–slight ICIQ-UI-SF	Moderate–severe ICIQ-UI-SF	
	n = 217	n = 125	
**Age (years)**	62 ± 13	68 ± 13	0.001
**Age groups**			<0.001
Age group 40–50 years	49 (23%)	22 (18%)	
Age group 51–64 years	79 (36%)	24 (19%)	
Age group ≥65 years	89 (41%)	79 (63%)	
**Education level**			<0.001
Primary level	48 (22%)	50 (40%)	
Secondary level	95 (44%)	51 (41%)	
Higher level	74 (34%)	24 (19%)	
**Occupational status**			0.002
Retired	95 (44%)	79 (63%)	
Sedentary work	86 (40%)	33 (27%)	
Manual work	36 (16%)	13 (10%)	
**Comorbidities**			
Arterial hypertension	90 (42%)	77 (62%)	<0.001
COPD/asthma	19 (9%)	8 (6%)	0.837
Diabetes type 2	20 (9%)	36 (29%)	<0.001
Atrial Fibrillation	10 (5%)	7 (6%)	0.684
Depression (on medication)	19 (9%)	14 (11%)	0.461
Rheumatologic disease	21 (10%)	15 (12%)	0.500
Hyperlipidemia	57 (26%)	51 (41%)	0.005

LUTS: Lower Urinary Tract Symptoms; ICIQ-UI-SF: International Consultation on Incontinence Questionnaire–Urinary Incontinence Short Form; COPD: Chronic Obstructive Pulmonary Disease.

## Data Availability

The datasets generated during and/or analyzed during the current study are available from the corresponding author upon reasonable request.

## References

[1] Gajewski J.B., Drake M.J. (2018). Neurological lower urinary tract dysfunction essential terminology. Neurourol. Urodyn..

[2] Sperry B.W., Summers S., Patel D., Garcia M., Bandeko C. (2021). A Practical Approach for Primary Care Practitioners to Evaluate and Manage Lower Urinary Tract Symptoms and Benign Prostatic Hyperplasia. Fed. Pract..

[3] Huang J., Chan C.K., Yee S., Deng Y., Bai Y., Chan S.C., Tin M.S., Liu X., Lok V., Zhang L. (2023). Global burden and temporal trends of lower urinary tract symptoms: A systematic review and meta-analysis. Prostate Cancer Prostatic Dis..

[4] Coyne K.S., Sexton C.C., Kopp Z.S., Ebel-Bitoun C., Milsom I., Chapple C. (2011). The impact of overactive bladder on mental health, work productivity and health-related quality of life in the UK and Sweden: Results from EpiLUTS. BJU Int..

[5] Choi E.P.H., Lam C.L.K., Chin W.Y. (2016). Mental health of Chinese primary care patients with lower urinary tract symptoms. Psychol. Health Med..

[6] Choi E.P.H.H., Lam C.L.K.K., Chin W.-Y.Y. (2014). The health-related quality of life of Chinese patients with lower urinary tract symptoms in primary care. Qual. Life Res..

[7] Coyne K.S., Wein A.J., Tubaro A., Sexton C.C., Thompson C.L., Kopp Z.S., Aiyer L.P. (2009). The burden of lower urinary tract symptoms: Evaluating the effect of LUTS on health-related quality of life, anxiety and depression: EpiLUTS. BJU Int..

[8] Choi E.P.H., Huang J., Chau P.H., Wan E.Y.F. (2021). Health-related quality of life among Chinese primary care patients with different lower urinary tract symptoms: A latent class analysis. Qual. Life Res..

[9] Roy H.A., Nettleton J., Blain C., Dalton C., Farhan B., Fernandes A., Georgopoulos P., Klepsch S., Lavelle J., Martinelli E. (2020). Assessment of patients with lower urinary tract symptoms where an undiagnosed neurological disease is suspected: A report from an International Continence Society consensus working group. Neurourol. Urodyn..

[10] Qasrawi H., Tabouni M., Almansour S.W., Ghannam M., Abdalhaq A., Abushamma F., Koni A.A., Zyoud S.H. (2022). An evaluation of lower urinary tract symptoms in diabetic patients: A cross-sectional study. BMC Urol..

[11] McVary K.T., Roehrborn C.G., Avins A.L., Barry M.J., Bruskewitz R.C., Donnell R.F., Foster H.E., Gonzalez C.M., Kaplan S.A., Penson D.F. (2011). Update on AUA guideline on the management of benign prostatic hyperplasia. J. Urol..

[12] Gacci M., Sakalis V.I., Karavitakis M., Cornu J.N., Gratzke C., Herrmann T.R.W., Kyriazis I., Malde S., Mamoulakis C., Rieken M. (2022). European Association of Urology Guidelines on Male Urinary Incontinence. Eur. Urol..

[13] Pesonen J.S., Vernooij R.W.M., Cartwright R., Aoki Y., Agarwal A., Mangera A., Markland A.D., Tsui J.F., Santti H., Griebling T.L. (2020). The impact of nocturia on falls and fractures: A systematic review and meta-analysis. J. Urol..

[14] Martin S.A., Haren M.T., Marshall V.R., Lange K., Wittert G.A., Members of the Florey Adelaide Male Ageing Study (2011). Prevalence and factors associated with uncomplicated storage and voiding lower urinary tract symptoms in community-dwelling Australian men. World J. Urol..

[15] Agarwal A., Eryuzlu L.N., Cartwright R., Thorlund K., Tammela T.L., Guyatt G.H., Auvinen A., Tikkinen K.A. (2014). What is the most bothersome lower urinary tract symptom? Individual- and population-level perspectives for both men and women. Eur. Urol..

[16] Abdelmoteleb H., Jefferies E.R., Drake M.J. (2016). Assessment and management of male lower urinary tract symptoms (LUTS). Int. J. Surg..

[17] Matsuda Y., Kobayashi K., Fukuta F., Takayanagi A., Hashimoto K., Tanaka T., Masumori N. (2021). Which Happens Earlier, Lower Urinary Tract Symptoms or Erectile Dysfunction?. Sex. Med..

[18] Wu J.M., Vaughan C.P., Goode P.S., Redden D.T., Burgio K.L., Richter H.E., Markland A.D. (2014). Prevalence and trends of symptomatic pelvic floor disorders in US women. Obset. Gynecol..

[19] Offermans M.P., Du Moulin M.F., Hamers J.P., Dassen T., Halfens R.J. (2009). Prevalence of urinary incontinence and associated risk factors in nursing home residents: A systematic review. Neurol. Urodyn..

[20] Anger J.T., Saigal C.S., Litwin M.S., Urologic Diseases of America Project (2006). The prevalence of urinary incontinence among community dwelling adult women: Results from the National Health and nutrition examination survey. J. Urol..

[21] Minassian V.A., Yan X., Lichtenfeld M.J., Sun H., Stewart W.F. (2012). The iceberg of health care utilization in women with urinary incontinence. Int. Urogynecol. J..

[22] Brown J.S., McGhan W.F., Chokroverty S. (2000). Comorbidities associated with overactive bladder. Am. J. Manag. Care.

[23] Wagner T.H., Hu T.W., Bentkover J., LeBlanc K., Stewart W., Corey R., Zhou Z., Hunt T. (2002). Health-related consequences of overactive bladder. Am. J. Manag. Care.

[24] Gibson W., Hunter K.F., Camicioli R., Booth J., Skelton D.A., Dumoulin C., Paul L., Wagg A. (2018). The association between lower urinary tract symptoms and falls: Forming a theoretical model for a research agenda. Neurol. Urodyn..

[25] Ninomiya S., Kawahara T., Tsutsumi S., Ito H., Makiyama K., Uemura H. (2023). Lower urinary tract symptoms are elevated with depression in Japanese women. Low. Urin. Tract Symptoms.

[26] Gibson W., Harari D., Husk J., Lowe D., Wagg A. (2016). A national benchmark for the initial assessment of men with LUTS: Data from the 2010 Royal College of Physicians National Audit of Continence Care. World J. Urol..

[27] Drake M.J., Worthington J., Frost J., Sanderson E., Cochrane M., Cotterill N., Fader M., McGeagh L., Hashim H., Macaulay M. (2023). Treatment of lower urinary tract symptoms in men in primary care using a conservative intervention: Cluster randomised controlled trial. BMJ.

[28] Basra R.K., Cortes E., Khullar V., Kelleher C. (2012). A comparison study of two lower urinary tract symptoms screening tools in clinical practice: The B-SAQ and OAB-V8 questionnaires. J. Obstet. Gynaecol..

[29] Gravas S., Gacci M., Gratzke C., Herrmann T.R.W., Karavitakis M., Kyriazis I., Malde S., Mamoulakis C., Rieken M., Sakalis V.I. (2023). Summary Paper on the 2023 European Association of Urology Guidelines on the Management of Non-Neurogenic Male Lower Urinary Tract Symptoms. Eur. Urol..

[30] Nambiar A.K., Arlandis S., Bø K., Cobussen-Boekhorst H., Costantini E., de Heide M., Farag F., Groen J., Karavitakis M., Lapitan M.C. (2022). European Association of Urology Guidelines on the Diagnosis and Management of Female Non-neurogenic Lower Urinary Tract Symptoms. Part 1: Diagnostics, Overactive Bladder, Stress Urinary Incontinence, and Mixed Urinary Incontinence. Eur. Urol..

[31] Lionis C., Vlachonikolis L., Bathianaki M., Daskalopoulos G., Anifantaki S., Cranidis A. (2000). Urinary incontinence, the hidden health problem of Cretan women: Report from a primary care survey in Greece. Women Health.

[32] Liapis A., Bakas P., Liapi S., Sioutis D., Creatsas G. (2010). Epidemiology of female urinary incontinence in the Greek population: EURIG study. Int. Urogynecol. J..

[33] Anifantaki S., Filiz T.M., Alegakis A., Topsever P., Markaki A., Cinar N.D., Sofras F., Lionis C. (2009). Does urinary incontinence affect quality of life of Greek women less severely? A cross-sectional study in two Mediterranean settings. Qual. Life Res..

[34] Barry M.J., Fowler F.J., O’Leary M.P., Bruskewitz R.C., Holtgrewe H.L., Mebust W.K., Cockett A.T. (1992). The measurement committee of the American urological association, the American Urological Association symptom index for benign prostatic hyperplasia. J. Urol..

[35] Abrams P., Chapple C., Khoury S., Roehrborn C., de la Rosette J., International Consultation on New Developments in Prostate Cancer and Prostate Diseases (2013). Evaluation and treatment of lower urinary tract symptoms in older men. J. Urol..

[36] Papaefstathiou E., Moysidis K., Sarafis P., Ioannidis E., Hatzimouratidis K. (2019). The impact of Diabetes Mellitus on Lower urinary tract symptoms (LUTS) in both male and female patients. Diabetes Metab. Syndr..

[37] Rosen R.C., Cappelleri J.C., Smith M.D., Lipsky J., Peña B.M. (1999). Development and evaluation of an abridged, 5-item version of the International Index of Erectile Function (IIEF-5) as a diagnostic tool for erectile dysfunction. Int. J. Impot. Res..

[38] Hatzimouratidis K., Tsimtsiou Z., Karantana A., Hatzichristou D. (2001). Cultural and linguistic validation of International Index of Erectile Function (IIEF) in Greek language. Hell. Urol..

[39] Avery K., Donovan J., Peters T.J., Shaw C., Gotoh M., Abrams P. (2004). ICIQ: A brief and robust measure for evaluating the symptoms and impact of urinary incontinence. Neurourol. Urodyn..

[40] Athanasiou S., Grigoriadis T., Kyriakidou N., Giannoulis G., Antsaklis A. (2012). The validation of international consultation on incontinence questionnaires in the Greek language. Neurourol. Urodyn..

[41] Zhang A.Y., Xu X. (2018). Prevalence, Burden, and Treatment of Lower Urinary Tract Symptoms in Men Aged 50 and Older: A Systematic Review of the Literature. SAGE Open Nurs..

[42] Chapple C., Castro-Diaz D., Chuang Y.C., Lee K.S., Liao L., Liu S.P., Wang J., Yoo T.K., Chu R., Sumarsono B. (2017). Prevalence of Lower Urinary Tract Symptoms in China, Taiwan, and South Korea: Results from a Cross-Sectional, Population-Based Study. Adv. Ther..

[43] Coyne K.S. (2009). The prevalence of lower urinary tract symptoms (LUTS) in the USA, the UK and Sweden: Results from the Epidemiology of LUTS (EpiLUTS) study. BJU Int..

[44] Irwin D.E., Milsom I., Kopp Z., Abrams P., Artibani W., Herschorn S. (2009). Prevalence, severity, and symptom bother of lower urinary tract symptoms among men in the EPIC study: Impact of overactive bladder. Eur. Urol..

[45] Soler R., Gomes C.M., Averbeck M.A., Koyama M. (2017). The prevalence of lower urinary tract symptoms (LUTS) in Brazil: Results from the epidemiology of LUTS (Brazil LUTS) study. Neurourol. Urodyn..

[46] Isa N.M.M., Aziz A.F.A. (2020). Lower Urinary Tract Symptoms: Prevalence and Factors Associated with Help-Seeking in Male Primary Care Attendees. Korean J. Fam. Med..

[47] Lai U.C., Wun Y.T., Luo T.C., Pang S.M. (2011). In a free healthcare system, why do men not consult for lower urinary tract symptoms (LUTS)?. Asia Pac. Fam. Med..

[48] Apostolidis A., Kirana P.S., Chiu G., Link C., Tsiouprou M., Hatzichristou D. (2009). Gender and age differences in the perception of bother and health care seeking for lower urinary tract symptoms: Results from the hospitalised and outpatients’ profile and expectations study. Eur. Urol..

[49] Taylor J., McGrother C.W., Harrison S.C., Assassa P.R., Leicestershire MRC Incontinence Study Team (2006). Lower urinary tract symptoms and related help-seeking behaviour in South Asian men living in the UK. BJU Int..

[50] Shaw C., Tansey R., Jackson C., Hyde C., Allan R. (2001). Barriers to help seeking in people with urinary symptoms. Fam. Pract..

[51] Apostolidis A., de Nunzio C., Tubaro A. (2012). What determines whether a patient with LUTS seeks treatment?: ICI-RS 2011. Neurourol. Urodyn..

[52] Malde S., Umbach R., Wheeler J.R., Lytvyn L., Cornu J.N., Gacci M., Gratzke C., Herrmann T.R.W., Mamoulakis C., Rieken M. (2021). A Systematic Review of Patients’ Values, Preferences, and Expectations for the Diagnosis and Treatment of Male Lower Urinary Tract Symptoms. Eur. Urol..

[53] Wolters R., Wensing M., van Weel C., van der Wilt G.J., Grol R.P. (2002). Lower urinary tract symptoms: Social influence is more important than symptoms in seeking medical care. BJU Int..

[54] Lammers H.A., van Wijnhoven R., Teunissen T.A., Harmsen S., Lagro-Janssen A.L. (2015). Why do men suffering from LUTS seek primary medical care? A qualitative study. J. Eval. Clin. Pract..

[55] Horrocks S., Somerset M., Stoddart H., Peters T.J. (2004). What prevents older people from seeking treatment for urinary incontinence?: A qualitative exploration of barriers to the use of community continence services. Fam. Pract..

[56] Kupelian V., Wei J.T., O’Leary M.P., Kusek J.W., Litman H.J., Link C.L., McKinlay J.B., BACH Survery Investigators (2006). Prevalence of lower urinary tract symptoms and effect on quality of life in a racially and ethnically diverse random sample: The Boston area community health (BACH) survey. Arch. Intern. Med..

[57] Derimachkovski G., Yotovski V., Mladenov V., Ianev K., Mladenov D. (2014). Men with LUTS and diabetes mellitus. Acta Chir. Iugosl..

[58] Coyne K.S., Kaplan S.A., Chapple C.R., Sexton C.C., Kopp Z.S., Bush E.N., Aiyer L.P. (2009). Risk factors and comorbid conditions associated with lower urinary tract symptoms: EpiLUTS. BJU Inter..

[59] Litman H.J., Steers W.D., Wei J.T., Kupelian V., Link C.L., McKinlay J.B., Boston Area Community Health Survey Investigators (2007). Relationship of lifestyle and clinical factors to lower urinary tract symptoms: Results from Boston Area Community Health survey. Urology.

[60] Vaughan C.P., Johnson T.M., Goode P.S., Redden D.T., Burgio K.L., Markland A.D. (2011). Vitamin D and lower urinary tract symptoms among US men: Results from the 2005–2006 National Health and Nutrition Examination Survey. Urology.

[61] Coyne K.S., Zhou Z., Bhattacharyya S.K., Thompson C.L., Dhawan R., Versi E. (2003). The prevalence of nocturia and its effect on health-related quality of life and sleep in a community sample in the USA. BJU Int..

[62] Li M.K., Garcia L.A., Rosen R. (2005). Lower urinary tract symptoms and male sexual dysfunction in Asia: A survey of ageing men from five Asian countries. BJU Int..

[63] Vartolomei L., Cotruș A., Tătaru S.O., Vartolomei M.D., Man A., Ferro M., Stanciu C., Sin A.I., Shariat S.F. (2022). Lower urinary tract symptoms are associated with clinically relevant depression, anxiety, and stress symptoms. Aging Male.

[64] Rosen R., Altwein J., Boyle P. (2003). Lower urinary tract symptoms and male sexual dysfunction: The multinational survey of the aging male (MSAM-7). Eur. Urol..

[65] Nakamura M., Fujimura T., Nagata M. (2012). Association between lower urinary tract symptoms and sexual dysfunction assessed using the core lower urinary tract symptom score and International Index of Erectile Function-5 questionnaires. Aging Male.

[66] Ponholzer A., Temml C., Obermayr R. (2004). Association between lower urinary tract symptoms and erectile dysfunction. Urology.

[67] Fwu C.W., Kirkali Z., McVary K.T. (2015). Cross-sectional and longitudinal associations of sexual function with lower urinary tract symptoms in men with benign prostatic hyperplasia. J. Urol..

[68] Apostolidis A., Rantell A., Anding R., Kirschner-Hermanns R., Cardozo L. (2017). How does lower urinary tract dysfunction (LUTD) affect sexual function in men and women? ICI-RS 2015-Part 2. Neurourol. Urodyn..

[69] Aleksandra R., Aleksandra S., Iwona R. (2022). Erectile Dysfunction in Relation to Metabolic Disorders and the Concentration of Sex Hormones in Aging Men. Int. J. Environ. Res. Public Health.

[70] Gonzalez-Sanchez B., Cendejas-Gomez J., Rivera-Ramirez J.A., Herrera-Caceres J.O., Olvera-Posada D., Villeda-Sandoval C.I., Castillejos-Molina R.A., Feria-Bernal G., Garcia-Mora A., Rodriguez-Covarrubias F. (2016). The correlation between lower urinary tract symptoms (LUTS) and erectile dysfunction (ED): Results from a survey in males from Mexico City (MexiLUTS). World J. Urol..

[71] Yafi F.A., Jenkins L., Albersen M., Corona G., Isidori A.M., Goldfarb S., Maggi M., Nelson C.J., Parish S., Salonia A. (2016). Erectile dysfunction. Nat. Rev. Dis. Primers.

[72] Fadzil M.A., Sidi H., Ismail Z., Hassan M.R., Thuzar K., Midin M., Nik Jaafar N.R., Das S. (2014). Socio-demographic and psychosocial correlates of erectile dysfunction among hypertensive patients. Compr. Psychiatry.

[73] Feng C., Yang Y., Chen L., Guo R., Liu H., Li C., Wang Y., Dong P., Li Y. (2022). Prevalence and Characteristics of Erectile Dysfunction in Obstructive Sleep Apnea Patients. Front. Endocrinol..

[74] Akgül M., Yazıcı C., Doğan Ç., Özcan R., Şahin M.F. (2021). Erectile dysfunction iceberg in an urology outpatient clinic: How can we encourage our patients to be more forthcoming?. Andrologia.

[75] Nihira M.A., Henderson N. (2003). Epidemiology of urinary incontinence in women. Curr. Womens Health Rep..

[76] Patel U.J., Godecker A.L., Giles D.L., Brown H.W. (2022). Updated prevalence of urinary incontinence in women: 2015–2018 national population-based survey data. Female Pelvic Med. Reconstr. Surg..

[77] Schüssler-Fiorenza Rose S.M., Gangnon R.E., Chewning B., Wald A. (2015). Increasing Discussion Rates of Incontinence in Primary Care: A Randomized Controlled Trial. J. Women’s Health.

[78] Cerruto M.A., D’Elia C., Aloisi A., Fabrello M., Artibani W. (2013). Prevalence, incidence and obstetric factors’ impact on female urinary incontinence in Europe: A systematic review. Urol. Int..

[79] Cooper J., Annappa M., Quigley A., Dracocardos D., Bondili A., Mallen C. (2015). Prevalence of female urinary incontinence and its impact on quality of life in a cluster population in the United Kingdom (UK): A community survey. Prim. Health Care Res. Dev..

[80] Lee U.J., Feinstein L., Ward J.B., Kirkali Z., Martinez-Miller E.E., Matlaga B.R., Kobashi K.C. (2021). Prevalence of urinary incontinence among a nationally representative sample of women, 2005–2016: Findings from the urologic diseases in America project. J. Urol..

[81] Dooley Y., Kenton K., Cao G., Luke A., Durazo-Arvizu R., Kramer H., Brubaker L. (2008). Urinary incontinence prevalence: Results from the National Health and Nutrition Examination Survey. J. Urol..

[82] Elenskaia K., Haidvogel K., Heidinger C., Doerfler D., Umek W., Hanzal E. (2011). The greatest taboo: Urinary incontinence as a source of shame and embarrassment. Wien. Klin. Wochenschr..

[83] Farage M.A., Miller K.W., Berardesca E., Maibach H.I. (2008). Psychosocial and societal burden of incontinence in the aged population: A review. Arch. Gynecol. Obstet..

[84] Pizzol D., Demurtas J., Celotto S., Maggi S., Smith L., Angiolelli G., Trott M., Yang L., Veronese N. (2021). Urinary incontinence and quality of life: A systematic review and meta-analysis. Aging Clin. Exp. Res..

[85] Worthington J., Frost J., Lane J.A., Robles L.A., Rees J., Taylor G., Drake M.J., Ridd M. (2021). Effective management of male lower urinary tract symptoms in primary care. Br. J. Gen. Pract..

[86] Di Bello F., Pezone G., Muzii B., Cilio S., Ruvolo C.C., Scandurra C., Mocini E., Creta M., Morra S., Bochicchio V. (2024). Lower urinary tract symptoms in young-middle aged males with a diagnosis of obstructive sleep apnea syndrome. Neurourol. Urodyn..

[87] Markland A., Bavendam T., Cain C., Neill Epperson C., Fitzgerald C.M., Yvette LaCoursiere D., Shoham D.A., Smith A.L., Sutcliffe S., Rudser K. (2024). Occupational groups and lower urinary tract symptoms: A cross-sectional analysis of women in the Boston Area Community Health Study. Neurourol. Urodyn..

[88] Peleg L.C., Rabinovitch D., Lavie Y., Rabbie D.M., Horowitz I., Fruchter E., Gruenwald I. (2022). Post-SSRI Sexual Dysfunction (PSSD): Biological Plausibility, Symptoms, Diagnosis, and Presumed Risk Factors. Sex. Med. Rev..

[89] Semczuk-Kaczmarek K., Płatek A.E., Szymański F.M. (2020). Co-treatment of lower urinary tract symptoms and cardiovascular disease—Where do we stand?. Cent. Eur. J. Urol..

